# Altered Regulatory B Cell Subsets in Children with Type 1 Diabetes Mellitus

**DOI:** 10.1155/2020/8935694

**Published:** 2020-07-21

**Authors:** Mohamed A. El-Mokhtar, Nahla M. Elsherbiny, Douaa Sayed, Duaa M. Raafat, Eman Askar, Almontaser Hussein, Mohamed A. Y. Abdel-Malek, Amira M. Shalaby

**Affiliations:** ^1^Department of Medical Microbiology and Immunology, Faculty of Medicine, Assiut University, Egypt; ^2^Department of Clinical Pathology, South Egypt Cancer Institute, Assiut University, Egypt; ^3^Department of Pediatrics, Faculty of Medicine, Assiut University, Egypt; ^4^Assiut University Children's Hospital, Faculty of Medicine, Assiut University, Egypt

## Abstract

B regulatory cells (Breg) refer to characteristic subsets of B cells that generally exert anti-inflammatory functions and maintain peripheral tolerance mainly through their ability to secrete interleukin-10 (IL10). Dysregulation in the function of Breg cells was reported in several autoimmune diseases. However, the relation between Breg and children with type 1 diabetes (T1D) is poorly understood. Thus, this study is aimed at determining whether Breg cells play a role in T1D in children or not, so we hypothesized that an altered phenotype of B cell subsets is associated with T1D in children. Children with T1D (*n* = 29) and control children with normal blood glucose levels (*n* = 14) were recruited. The percentages of different circulating IL10-producing Breg subsets, including B10, immature transitional, and plasmablasts were determined using flow cytometry analysis. Furthermore, the association between different IL10-producing B cells and patient parameters was investigated. The percentage of circulating IL10^+^CD24^hi^CD27^+^ (B10) and IL10^+^CD24^hi^CD38^hi^ (immature transitional) subsets of Breg cells was significantly lower in T1D patients than in healthy controls. Moreover, these cells were also negatively correlated with fasting blood glucose and HbA1c levels. Breg cells did not correlate with autoantibody levels in the serum. These findings suggest that certain Breg subsets are numerically deficient in children with T1D. This alteration in frequency is associated with deficient islet function and glycemia. These findings suggest that Breg cells may be involved in the loss of auto-tolerance and consequent destruction of pancreatic cells and could, therefore, be a potential target for immunotherapy.

## 1. Introduction

Type 1 diabetes (T1D) is a common chronic autoimmune disease that attacks children predominantly and persists for life. For unclear reasons, the incidence is steadily increasing in children younger than 15 years [1]. Such patients are characterized by the destruction of insulin-producing *β* cells leading to insulin deficiency and hyperglycemia. Uncontrolled patients are also subjected to long-term complications [2] [3]. The management of this disease remains an overwhelming challenge requiring insulin analog regimens, blood glucose monitoring, and controlling carbohydrate intake [4]. Diabetic patients are in a strong need for a curative therapy that avoids the exogenous insulin administration. Proper understanding of the disease pathogenesis may help in developing new therapeutic strategies that improve the control and prevent the complications associated with T1D.

To date, several overlapping phenotypes of Breg cells have been identified [5]. Among these subsets are the B10 cells (CD24^hi^CD27^+^) which are known to suppress monocyte inflammatory functions including TNF*α* production [6], immature or transitional B cells (CD24^hi^CD38^hi^) which decrease IFN*γ* and TNF production [7], and the plasmablasts (CD38^hi^CD27^+^) which were reported to suppress the DC ability to generate pathogenic CD4^+^ T cells in a mouse model of experimental autoimmune encephalomyelitis [8]. There is no specific lineage marker for Breg cells, but they are differentiated according to the expression of certain surface markers. However, a common distinguishing character of these cells is the production of IL10 that mediates the immunosuppressive functions of these cells [9].

Although type 1 diabetes (T1D) has been classically described as a CD4+ T cell-mediated disease, yet B cells also play an essential role in the autoimmune destruction of pancreatic *β* cells [10]. Therefore, B cell-depleting therapy was developed for treating T1D. However, extended clinical trials of these experiments showed that B cell depletion did not markedly alter the underlying pathophysiology of the disease [11]. A possible explanation for the unsatisfactory results of the B-lymphocyte-directed therapies is the coremoval of the beneficial Breg cells that participate in the maintenance of self-tolerance against autoimmune diabetes [12].

Dysregulation of Breg cells was reported in several autoimmune diseases, including rheumatoid arthritis (RA), systemic lupus erythematosus (SLE), and multiple sclerosis [13, 14]. However, little is known about the role of Bregs in children with T1D. Therefore, the aim of this study was to compare the changes in different IL10-producing Breg subsets in children with T1D to healthy controls.

## 2. Material and Methods

### 2.1. Ethics Statement

The study was approved by the Ethics Committee of the Faculty of Medicine, Assiut University, and was conducted in accordance with the provisions of the Declaration of Helsinki. Informed written consent for sample collection and research was obtained from parents of children before enrolment in the study.

### 2.2. Study Subjects and Clinical Parameters

The study was carried out in the period from mid-2018 to mid-2019. During this period, 29 children with T1D and 14 age- and sex-matched controls were enrolled in the study, and their parents provided written consent. Children were excluded from the study if they had other infections and/or autoimmune diseases based on the preliminary clinical investigations. Children were admitted to the endocrine unit, Pediatrics Assiut University Hospital, Assiut, Egypt. Diabetes was diagnosed according to World Health Organization (WHO) criteria [15]. Blood samples were taken from patients and controls for the estimation of glucose and HbA1c levels. For those who were confirmed to be diabetic, autoantibodies were measured. Concerning the treatment regimen, diabetic children received a basal and bolus regimen, in which rapid onset insulin was given with meals with a slow-onset, long-duration background. Insulin was given once at bedtime. Healthy control children had normoglycemia and normal HbA1c levels.

HbA1c was measured by automated liquid chromatography (VARIANT II Hemoglobin Testing System; Bio-Rad Laboratories, Hercules, CA). Auto-antibodies to Zinc transporter-8 (ZnT8), glutamic acid decarboxylase antibodies (GADA), and insulinoma-2 antigen antibodies (IA2A) were estimated using commercial ELISA kits (DLD Diagnotika, GMBH, Germany). Body mass index (BMI) was calculated as weight (kg)/squared height (m). BMI percentiles were determined from the Centers for Disease Control and Prevention growth curves [16].

### 2.3. Preparation of PBMC and Flow Cytometry Analysis

**Figure 1:**
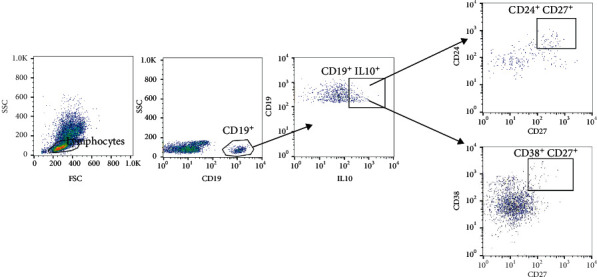
Gating strategy used to identify different peripheral B cell subsets. The initial CD19^+^ gate was derived from a lymphocyte gate (defined on SSC and FSC) followed by dot plots gated on IL10^+^CD19^+^ B cells showing the indicated B cell subsets.

Peripheral blood mononuclear cells were separated from whole blood using Ficoll-Pague solution (Sigma Aldrich, Germany), plated at a density of 1 × 10^6^ cells/ml in complete RPMI 1640 medium supplemented with 10% fetal bovine serum, 1 mM sodium pyruvate, penicillin, streptomycin, 4 mM L-glutamine, and 0.1% 2-mercaptoethanol (all from Gibco) in the presence of phorbol myristate acetate (50 ng/ml; Sigma-Aldrich), ionomycin (1 *μ*g/ml; Sigma-Aldrich), and brefeldin A (BioLegend) in 12-well plates for 18 h, at 37°C. For analysis of different Breg subsets, cells were surface stained with the following anti-human mAb (all from BioLegend): CD19 APC, CD24 FITC, CD38 PerCP-Cy5.5, and CD27 PE. For intracellular IL10 staining, cells were fixed and permeabilized using the Fixation/Permeabilization kit (BD Biosciences) and stained with anti-human IL10 PE-Cy7. Stained cells were acquired by FACS Canto II flow cytometer (BD Bioscience), and data were analyzed using FlowJo software 7.6.1 (Tree Star Inc., USA). The gating strategy used for B cell phenotyping is shown in Figure 1. The following B cell subsets were identified: CD19+ (total B cells), B10 cells (CD24^hi^CD27^+^), immature transitional B cells (CD24hiCD38hi), and plasmablasts (CD38^hi^CD27^+^).

### 2.4. Statistical Analysis

Statistical analyses were performed using SPSS version 19.0 (IBM Corporation, Chicago, IL). Data are presented as mean ± SD. Associations of the frequencies of Breg subsets with other clinical parameters were estimated by Pearson test or Spearman nonparametric correlations. Comparison between two groups was carried out using unpaired *t*-test. The Mann-Whitney test (*U*) was used to compare two groups of nonparametric data. Differences were considered significant at *P* < 0.05.

## 3. Results

Twenty-nine children with T1D with a median age of 7 (3.4-11) years and 14 age- and sex-matched control children with normal glucose levels were enrolled in the current study.  Table 1 summarizes the features of the subjects in each group. Diabetic patients included 14 males and 15 female children. The median age at diagnosis was 4.5 (2-6.5) years, while the median duration of T1D after diagnosis was 1.6 (0.1-4.85) years. Children with T1D were slightly lighter and shorter than the control group.

**Figure 2:**
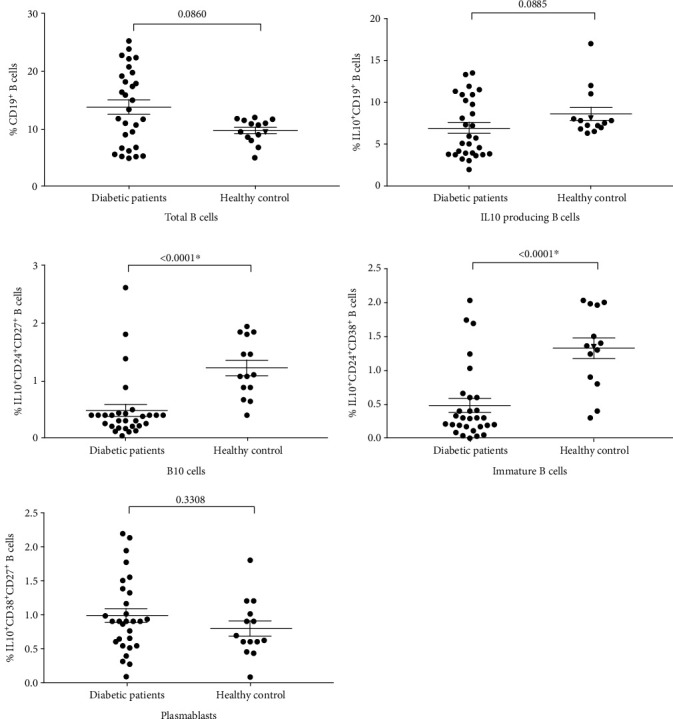
Alterations in the frequencies of different B cell subset in children with T1D compared to control subjects. Horizontal lines show means ± standard error. ∗ indicate significance (*P* < 0.05).

**Figure 3:**
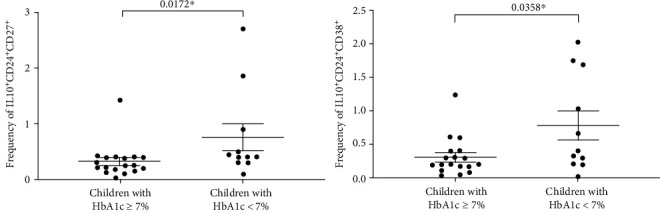
Frequency of B10 and immature transitional Breg subsets according to children's HbA1c levels. Horizontal lines show means ± standard error. ∗ indicate significance (*P* < 0.05).

The difference in frequencies of different Breg cell phenotypes in children with diabetes is summarized in Figure 2. We did not observe any significant difference in the frequency of total B cells (CD19^+^) or total IL10-secreting B cells (IL10^+^CD19^+^) between T1D patients and controls (14 ± 6.6 and 6.9 ± 3.6 in T1D patients vs. 9.7 ± 2.1 and 8.6 ± 2.9 in controls for B cells and IL10-secreting B cells, respectively). Also, no significant difference in IL10^+^CD38^hi^CD27^+^ subset of B cells was observed in diabetic children compared to the controls (0.98 ± 0.55 in patients and 0.79 ± 0.42 in controls). Interestingly, IL10^+^CD24^hi^CD27^+^ (B10 cells) and IL10^+^CD24^hi^CD38^hi^ (immature transitional) cells were significantly lower in diabetic children than in healthy controls. The mean values of IL10^+^CD24^hi^CD27^+^ and IL10^+^CD24^hi^CD38^hi^ B cells in patients were 0.49 ± 0.57 and 0.48 ± 0.54 while those in controls were 1.3 ± 0.53 and 1.3 ± 0.57, respectively. Since the frequency of B10 and immature transitional subsets were modulated in the diabetic children, we compared the distribution of these 2 subsets in children with HbA1c ≥7% and those with HbA1c less than 7%. Children with HbA1c ≥7% levels had significantly lower levels of IL10^+^CD24^hi^CD27^+^ (B10 cells) and IL10^+^CD24^hi^CD38^hi^ (immature transitional) compared to children with HbA1c <7% (Figure 3).

Given the significant association of T1D with the certain assessed B cell phenotypes, we next performed a linear regression analysis to analyze whether the changes in IL10-producing Breg subsets had any association with the clinical parameters. IL10-producing B10 and immature transitional B cells were negatively correlated with fasting blooding glucose (*r* = −0.132 and 0.124; *P* values = 0.01^∗^ for both). Of note, HbA1c level also was negatively correlated with IL10-producing B cells, B10 cells, and immature transitional B cells (*r* = −0.168, -0.135, and -0.1587; *P* values = 0.02^∗^, 0.03^∗^, and 0.02^∗^, respectively). Also, C-peptide correlated with B10 cells (*r* = 0.146; *P* value = 0.02^∗^). We found no significant association between any of the studied autoantibodies with the frequency of Breg cell subsets (Table 2).

## 4. Discussion

B cells and T cells play important roles in the pathogenesis of many infections and autoimmune diseases [17]. In the present study, we employed a flow cytometry approach to characterize different IL10-producing Breg subsets in the peripheral blood of T1D children and healthy individuals to assess whether these cell subsets are implicated in the regulation of this immune-mediated disease. We hypothesized that an altered B cell phenotype is associated with autoimmune diabetes. Although it is appreciated that multiple distinct Breg subsets are described, this study is specifically focused on the changes in IL10-producing B10, immature transitional, and plasmablast subsets of regulatory B cells. The major finding of this study was a distinct alteration of B10 and immature transitional Breg subsets across the diabetes spectrum and their association with some laboratory parameters.

We did not observe a distinct alteration in the overall frequency of total peripheral CD19+ B cells or total IL10-producing B cells in children with T1D compared to controls or any association with the demographic or laboratory parameters, which is consistent with other published studies [18, 19]. Importantly to note, children with T1D had lower B10 and immature transitional subsets of Breg cells than control subjects. Consistent with this finding, children with bad glycemic control, evidenced by HbA1c ≥7%, showed lower frequencies of these Breg subsets than those with HbA1c <7%, giving a further confirmation to the role of Breg in T1D pathogenesis. Similar to our results, Deng et al. [20] reported that patients with T1D had very low levels of interleukin-10-producing regulatory B10 cells even lower than patients with T2D or those with latent autoimmune diabetes in adults. B10 cells were shown to decrease the activation of T cells by lowering surface MHCII and costimulatory molecules and decrease Ag presentation by DC and suppression of Th17 responses [21]. They also decrease inflammatory cytokine production by monocytes [6]. In animal experiments, they protected the glomerular endothelial cells and attenuated the progression on lupus nephritis [22].

Similarly, immature Breg cells (CD24^hi^CD38^hi^) were reported to decrease IFN*γ* and TNF production by TH1 and IL17 by TH17 cells and suppress CD8^+^ T cell responses. They also induced regulatory T cells and maintained iNKT cells [7, 23]. Given the negative correlation between B10 cells and fasting blood glucose and HbA1c and the positive correlation with C-peptide in one hand and the negative correlation between immature Breg cells and fasting blood glucose and HbA1c on the other, it is possible to elucidate that the decrease in B10 and immature B cells may promote the destruction of pancreatic islet cells. Also, the exact mechanism for the contribution of these Breg subsets in the breakdown of self-tolerance leading to pancreatic cell destruction is unknown and needs further functional studies. Interestingly, untreated rheumatoid arthritis, systemic sclerosis, and SLE patients had lower IL10 production by Breg cells than treated patients [24]. Of note, IL10-producing plasmablasts were not altered in T1D. Thus, these cells may appear to be less important in the establishment of pancreatic autoimmunity.

Bregs cells have been recently described as an essential immune system component that exhibits downregulatory function by suppressing the adaptive and innate arms of the immune system, inflammation reactions, and autoimmune diseases, mainly through the secretion of IL10 [25]. The suppressive functions mediated by IL10 have been demonstrated both *in vitro* and *in vivo* in adaptive transfer assays [26]. In addition, the IL10-dependent suppressive role for the Breg cells has been proven in different models of autoimmune diseases, including SLE, rheumatoid arthritis, and multiple sclerosis [27–29]. Lu et al. [30] demonstrated that CD24^hi^CD38^hi^ B cells are potent suppressors for the differentiation of T helper 1 cells upon CD40L stimulation. However, Iwata et al. characterized human IL10-producing CD24^hi^CD27^+^ Breg cells and showed that they suppressed the production of CD4^+^TNF*α*^+^ T cells through an IL10-independent manner [6].

Consistent with the reported negative regulatory function of the Breg cells, significantly lower levels of IL10^+^ Breg cells were observed in rheumatoid arthritis patients. Interestingly, these Breg cells were functionally impaired and could not suppress the production of IFN*γ* by CD4^+^ T cells [31]. In an experimental study, Wu et al. [32] reported that IL10^+^ B cells ameliorated myocardial infarction-induced inflammation.

Contrary to our results, Thompson et al. [18] did not observe a difference in frequencies of IL10-producing Breg subsets between T1D patients and controls. The discrepancies might be a result of the difference in the age and geographical distribution of the study subjects.

The role of Breg cells in the immunopathogenesis of autoimmune diabetes is increasingly evident. In a mouse model for T1D, B cells infiltrated the islets of young NOD mice and initiated the destruction of the pancreatic *β* cells by diabetogenic T cells [33]. Serreze et al. [34] showed that NODJg mu(null) mice, in which B cells were genetically deleted, were resistant to autoimmune diabetes mellitus. B cells are largely responsible for the development of T1D because they act as antigen-presenting cells that enhance the expansion of the diabetogenic CD4^+^ T cells. Furthermore, autoantibodies specific for islet cell proteins are regularly produced by these cells and mediate the early steps of pathogenesis to beta cells [35].

When B cells were depleted in NOD mice using anti-CD20 mAb, hyperglycemia was successfully reversed in the early period [36, 37]. This promising preclinical result spurred the development of therapies targeting B cells for the treatment of T1D (Herold et al., 2005; 2002). However, pan-B cell targeting using anti-CD20 mAb did not lead to the expected successful outcomes in extended follow-up clinical trials. Indeed, such unsatisfactory results are attributed to the absence of selective targeting for the autoreactive B cells [11]. Such pan-B cell targeting will also result in reducing the IL10-producing Breg cells, which are highly needed due to their ability to regulate the self-tolerance and their characteristic anti-inflammatory effects [38]. Given the problems associated with the pan-B cell deletion, new alternative strategies are developed, such as targeting FasL which has been shown to specifically stop the development of T1D in animal models [39]. Therefore, our observations, which showed a reduction in Breg subsets in T1D patients, provide further support for the hypothesis that selective depletion of the autoreactive B cells without affecting regulatory subsets may improve the clinical use of B cell-targeted therapy for T1D.

We further investigated the correlation between different Breg subsets and some laboratory parameters. We found a negative correlation between pan IL10-producing B cells, B10 cells, immature B cells, and fasting blood glucose. Also, B10 cells and immature B cells positively correlated with C-peptide levels, which is in accordance with a previously published study [20]. However, contrary to others, we did not find a significant correlation regarding the frequency of Breg cells with age, sex, or autoantibody levels [40].

In summary, the current study describes a distinct alteration in the Breg subsets in children with T1D, which is associated with hyperglycemia. The current data is consistent with the inflammatory reaction in the pancreas, which is associated with autoimmune destruction of beta cells. Our results highlight the importance of selective B cell-targeted therapy for treating patients with T1D to selectively dampen the pathogenic B cells without affecting the beneficial anti-inflammatory Breg cells.

One limitation of our study is the relatively small number of study subjects. Another limitation is that the study was cross-sectional and was carried out at one-time point over a period of time. We did not follow up our cases longitudinally to see whether correction of their blood glucose levels will be associated with parallel changes in their Breg frequencies. Following up the patients will provide useful information about the changes in Breg subsets and glycemic control. Future studies should be planned to investigate the suppressive functions of Breg cells isolated from T1D children. It is possible that not only is the frequency of the Breg cells impaired but there also may be functional defects of Breg cells in T1D in children.

## Figures and Tables

**Table 1:** Clinical and demographic features of study subjects^∗^.

Variable	Type 1 diabetic patients (*n* = 29)	Controls (*n* = 14)
Sex (*n*)		
Male (%)	14 (48.2%)	6 (42.8%)
Female (%)	15 (51.7%)	8 (57.1%)
BMI percentile, mean ± SD	15 ± 1	16.5 ± 1
Weight (kg), mean ± SD	22 ± 10	26.5 ± 11.5
Height (cm), mean ± SD	115.5 ± 23.5	121 ± 23
Age (years), median (IQR)	7 (3.4-11)	7 (2.6-8.5)
Age at diagnosis (years), median (IQR)	4.5 (2-6.5)	NA
Duration of diabetes (years), median (IQR)	1.6 (0.1-4.85)	NA
Fasting blood glucose level (mg/dl), median (IQR)	320 (221-421)	NA
HbA_1c_ (%)	8 ± 2.2	4.9 ± 0.4
ZnT8A	11/29 (37.9%)	NA
GADA	23/29 (79.3%)	NA
IA2A	7/29 (24.1%)	NA

^∗^Data are expressed as mean ± standard deviation for parametric data, median (interquartile ranges) for nonparametric data, or *n* (%) for qualitative data. IQR: interquartile ranges; NA: not applicable.

**Table 2:** Correlations between B cell phenotypes and demographic, clinical and laboratory characteristics of the study patients.

Variable	B cell phenotype									
	CD19^+^ total B cells		IL10^+^CD19^+^ (IL10 B cells)		IL10^+^CD24^hi^CD27^+^ (B10)		IL10^+^CD24^hi^CD38^hi^ (immature B cells)		IL10^+^CD38^hi^CD27^+^ (plasmablast)	
	*r*	*P* value	*r*	*P* value	*r*	*P* value	*r*	*P* value	*r*	*P* value
Sex	-0.024	0.24	-0.018	0.40	-0.018	0.38	-0.014	0.87	0.017	0.35
Age	-0.043	0.09	-0.091	0.56	-0.370	0.09	-0.019	0.62	-0.084	0.45
Fasting blood glucose	-0.092	0.13	-0.041	0.08	-0.132	0.01^∗^	-0.124	0.01^∗^	-0.022	0.19
C-peptide	-0.013	0.80	-0.086	0.23	0.146	0.02^∗^	-0.01	0.42	-0. 017	0.26
HbA_1c_ (%)	0.131	0.11	-0.168	0.02^∗^	-0.135	0.03^∗^	-0.1587	0.02^∗^	-0.056	0.41
ZnT8A	0.043	0.09	0.027	0.42	0.038	0.12	-0.025	0.91	0.038	0.23
GADA	0.092	0.13	0.017	0.28	0.01	0.78	0.017	0.44	0.024	0.54
IA2A	0.013	0.80	0.012	0.21	0.031	0.36	-0.045	0.06	0.054	0.23

∗ indicate significance (*P* <0.05).

## Data Availability

The data used to support the findings of this study are available from the corresponding author upon request.

## Abbreviations

T1D: Type 1 diabetes
HbA1c: Hemoglobin A1c.

## References

[1] Patterson C. C., Gyürüs E., Rosenbauer J. (2012). Trends in childhood type 1 diabetes incidence in Europe during 1989-2008: evidence of non-uniformity over time in rates of increase. *Diabetologia*.

[2] Bluestone J. A., Herold K., Eisenbarth G. (2010). Genetics, pathogenesis and clinical interventions in type 1 diabetes. *Nature*.

[3] Deshpande A. D., Harris-Hayes M., Schootman M. (2008). Epidemiology of diabetes and diabetes-related complications. *Physical Therapy*.

[4] Harjutsalo V., Forsblom C., Groop P. H. (2011). Time trends in mortality in patients with type 1 diabetes: nationwide population based cohort study. *BMJ*.

[5] Yanaba K., Bouaziz J. D., Haas K. M., Poe J. C., Fujimoto M., Tedder T. F. (2008). A regulatory B cell subset with a unique CD1d^hi^CD5^+^ phenotype controls T cell-dependent inflammatory responses. *Immunity*.

[6] Iwata Y., Matsushita T., Horikawa M. (2011). Characterization of a rare IL-10-competent B-cell subset in humans that parallels mouse regulatory B10 cells. *Blood*.

[7] Mauri C., Blair P. A. (2010). Regulatory B cells in autoimmunity: developments and controversies. *Nature Reviews Rheumatology*.

[8] Matsumoto M., Baba A., Yokota T. (2014). Interleukin-10-producing plasmablasts exert regulatory function in autoimmune inflammation. *Immunity*.

[9] Lin W., Cerny D., Chua E. (2014). Human regulatory B cells combine phenotypic and genetic hallmarks with a distinct differentiation fate. *Journal of Immunology*.

[10] Fiorina P., Vergani A., Dada S. (2008). Targeting CD22 reprograms B-cells and reverses autoimmune diabetes. *Diabetes*.

[11] Pescovitz M. D., Greenbaum C. J., Bundy B. (2014). B-lymphocyte depletion with rituximab and beta-cell function: two-year results. *Diabetes Care*.

[12] Lykken J. M., Candando K. M., Tedder T. F. (2015). Regulatory B10 cell development and function. *International Immunology*.

[13] Correale J., Farez M., Razzitte G. (2008). Helminth infections associated with multiple sclerosis induce regulatory B cells. *Annals of Neurology*.

[14] Blair P. A., Noreña L. Y., Flores-Borja F. (2010). CD19^+^CD24^hi^CD38^hi^ B cells exhibit regulatory capacity in healthy individuals but are functionally impaired in systemic lupus erythematosus patients. *Immunity*.

[15] Alberti K. G., Zimmet P. Z. (1998). Definition, diagnosis and classification of diabetes mellitus and its complications. Part 1: diagnosis and classification of diabetes mellitus provisional report of a WHO consultation. *Diabetic Medicine*.

[16] Kuczmarski R. J., Ogden C. L., Grummer-Strawn L. M. (2000). CDC growth charts: United States. *Advance Data*.

[17] el-Mokhtar M. A., Elgendy S. G., Eldin A. S. (2020). Hepatitis C virus affects tuberculosis-specific T cells in HIV-negative patients. *Viruses*.

[18] Thompson W. S., Pekalski M. L., Simons H. Z. (2014). Multi-parametric flow cytometric and genetic investigation of the peripheral B cell compartment in human type 1 diabetes. *Clinical and Experimental Immunology*.

[19] Rodacki M., Svoren B., Butty V. (2006). Altered natural killer cells in type 1 diabetic patients. *Diabetes*.

[20] Deng C., Xiang Y., Tan T. (2016). Altered peripheral B-lymphocyte subsets in type 1 diabetes and latent autoimmune diabetes in adults. *Diabetes Care*.

[21] Gu Y., Yang J., Ouyang X. (2008). Interleukin 10 suppresses Th17 cytokines secreted by macrophages and T cells. *European Journal of Immunology*.

[22] Yu M., Song Y., Zhu M. X. (2015). B10 cells ameliorate the progression of lupus nephritis by attenuating glomerular endothelial cell injury. *Cellular Physiology and Biochemistry*.

[23] Flores-Borja F., Bosma A., Ng D. (2013). CD19^+^CD24^hi^CD38^hi^ B cells maintain regulatory T cells while limiting T_H_1 and T_H_17 differentiation. *Science Translational Medicine*.

[24] Llorente L., Richaud-Patin Y., Fior R. (1994). In vivo production of interleukin-10 by non-T cells in rheumatoid arthritis, Sjogren's syndrome, and systemic lupus erythematosus. *Arthritis and Rheumatism*.

[25] Rosser E. C., Mauri C. (2015). Regulatory B cells: origin, phenotype, and function. *Immunity*.

[26] Candando K. M., Lykken J. M., Tedder T. F. (2014). B10 cell regulation of health and disease. *Immunological Reviews*.

[27] Matsushita T., Horikawa M., Iwata Y., Tedder T. F. (2010). Regulatory B cells (B10 cells) and regulatory T cells have independent roles in controlling experimental autoimmune encephalomyelitis initiation and late-phase immunopathogenesis. *Journal of Immunology*.

[28] Carter N. A., Rosser E. C., Mauri C. (2012). Interleukin-10 produced by B cells is crucial for the suppression of Th17/Th1 responses, induction of T regulatory type 1 cells and reduction of collagen-induced arthritis. *Arthritis Research & Therapy*.

[29] Ben-Nun A., Kaushansky N., Kawakami N. (2014). From classic to spontaneous and humanized models of multiple sclerosis: impact on understanding pathogenesis and drug development. *Journal of Autoimmunity*.

[30] Lu Y., Liu F., Li C., Chen Y., Weng D., Chen J. (2017). IL-10-producing B cells suppress effector T cells activation and promote regulatory T cells in crystalline silica-induced inflammatory response in vitro. *Mediators of Inflammation*.

[31] Bankó Z., Pozsgay J., Szili D. (2017). Induction and differentiation of IL-10-producing regulatory B cells from healthy blood donors and rheumatoid arthritis patients. *Journal of Immunology*.

[32] Wu L., Dalal R., Cao C. D. (2019). IL-10-producing B cells are enriched in murine pericardial adipose tissues and ameliorate the outcome of acute myocardial infarction. *Proceedings of the National Academy of Sciences of the United States of America*.

[33] Diana J., Simoni Y., Furio L. (2013). Crosstalk between neutrophils, B-1a cells and plasmacytoid dendritic cells initiates autoimmune diabetes. *Nature Medicine*.

[34] Serreze D. V., Fleming S. A., Chapman H. D., Richard S. D., Leiter E. H., Tisch R. M. (1998). B lymphocytes are critical antigen-presenting cells for the initiation of T cell-mediated autoimmune diabetes in nonobese diabetic mice. *Journal of Immunology*.

[35] Silveira P. A., Grey S. T. (2006). B cells in the spotlight: innocent bystanders or major players in the pathogenesis of type 1 diabetes. *Trends in Endocrinology and Metabolism*.

[36] Hu C. Y., Rodriguez-Pinto D., du W. (2007). Treatment with CD20-specific antibody prevents and reverses autoimmune diabetes in mice. *The Journal of Clinical Investigation*.

[37] Xiu Y., Wong C. P., Bouaziz J. D. (2008). B lymphocyte depletion by CD20 monoclonal antibody prevents diabetes in nonobese diabetic mice despite isotype-specific differences in Fc*γ*R effector functions. *Journal of Immunology*.

[38] Hamad A. R., Ahmed R., Donner T., Fousteri G. (2016). B cell-targeted immunotherapy for type 1 diabetes: what can make it work?. *Discovery Medicine*.

[39] Hamad A. R., Arcara K., Uddin S., Donner T. (2012). The potential of Fas ligand (apoptosis-inducing molecule) as an unconventional therapeutic target in type 1 diabetes. *Frontiers in Immunology*.

[40] Morbach H., Eichhorn E. M., Liese J. G., Girschick H. J. (2010). Reference values for B cell subpopulations from infancy to adulthood. *Clinical and Experimental Immunology*.

